# Retraction Note: Thiazole-valine peptidomimetic (TTT-28) antagonizes multidrug resistance *in vitro* and *in vivo* by selectively inhibiting the efflux activity of ABCB1

**DOI:** 10.1038/s41598-025-33613-3

**Published:** 2025-12-30

**Authors:** Yi-Jun Wang, Bhargav A. Patel, Nagaraju Anreddy, Yun-Kai Zhang, Guan-Nan Zhang, Saeed Alqahtani, Satyakam Singh, Suneet Shukla, Amal Kaddoumi, Suresh V. Ambudkar, Tanaji T. Talele, Zhe-Sheng Chen

**Affiliations:** 1 Department of Pharmaceutical Sciences, College of Pharmacy and Health Sciences, St. John’s University, Queens, NY 11439 USA; 2 Department of Basic Pharmaceutical Sciences, School of Pharmacy, The University of Louisiana at Monroe, Monroe, LA 71201 USA; 3 Laboratory of Cell Biology, Center for Cancer Research, National Cancer Institute, National Institutes of Health, Bethesda, Maryland, MD 20892 USA

Retraction of: *Scientific Reports* 10.1038/srep42106, published online 09 February 2017

The Editors have retracted this Article.

After publication, concerns were raised regarding highly similar images within the Article and with another publication from the same authors^1^. Specifically:

- the H&E image in the Paclitaxel + TTT-28 in Figure 6B of this article appears to overlap the H&E image in the DOX in Figure 6B of^1^;
- the H&E image in the Vehicle in Figure 6B of this article appears to overlap the H&E image in the Vehicle in Figure 6A of^1^;
- the H&E image in the TTT-28 in Figure 6C of this article appears to overlap the H&E image in the Vehicle in Figure 6B of^1^;
- the ABCB1 image in the Paclitaxel in Figure 6C of this article appears to overlap the ABCB1 image in the DOX-TNP in Figure 6B of^1^;
- the Active Caspase-3 image in the Vehicle in Figure 6C of this article appears to overlap the Caspase-3 image in the Vehicle in Figure 6B of^1^;
- the Active Caspase-3 image in the TTT-28 in Figure 6C of this article appears to overlap the Caspase-3 image in the TNP in Figure 6B of^1^;
- the Active Caspase-3 image in the TTT-28 in Figure 6B of this article appears to overlap the Caspase-3 image in the Vehicle in Figure 6A of^1^;
- the Cleaved PARP-1 image in the TTT-28 in Figure 6B of this article appears to overlap the PARP image in the TNP in Figure 6A of^1^;
- the Cleaved PARP-1 image in the Vehicle in Figure 6B of this article appears to overlap the PARP image in the TNP in Figure 6A of^1^;
- the Cleaved PARP-1 image in the Vehicle in Figure 6C in this article appears to overlap PARP image in TNP in Figure 6B in^1^;
- the H&E image in the Vehicle in Figure 6B in this article appears to overlap the H&E image in the TTT-28 in Figure 6B of this article.

The Authors confirmed similarities but were unable to provide an adequate explanation to the concerns raised. The Editors therefore no longer have confidence in the presented data.

Additionally, according to the data presented in Figures 3A and 4A, the tumour burden in mice exceeded the limits stated in the NIH Guidelines for Humane Endpoints in Animal Study Proposals.

Yi-Jun Wang, Bhargav A. Patel, Yun-Kai Zhang, Guan-Nan Zhang, Suresh V. Ambudkar, Tanaji T. Talele and Zhe-Sheng Chen disagree with the retraction. Nagaraju Anreddy, Satyakam Singh, Suneet Shukla, and Amal Kaddoumi did not respond to the correspondence from the Editors about this retraction. The Editors have not been able to obtain a current email address for author Saeed Alqahtani.
